# m^6^A Regulator-Mediated Methylation Modification Model Predicts Prognosis, Tumor Microenvironment Characterizations and Response to Immunotherapies of Clear Cell Renal Cell Carcinoma

**DOI:** 10.3389/fonc.2021.709579

**Published:** 2021-07-06

**Authors:** Wenhao Xu, Xi Tian, Wangrui Liu, Aihetaimujiang Anwaier, Jiaqi Su, Wenkai Zhu, Fangning Wan, Guohai Shi, Gaomeng Wei, Yuanyuan Qu, Hailiang Zhang, Dingwei Ye

**Affiliations:** ^1^ Department of Urology, Fudan University Shanghai Cancer Center, Shanghai, China; ^2^ Department of Oncology, Shanghai Medical College, Fudan University, Shanghai, China; ^3^ Department of Urology, Affiliated Hospital of Youjiang Medical College for Nationalities, Baise, China

**Keywords:** clear cell renal cell carcinoma, tumor microenvironment, N^6^-methyladenosine, m^6^A modification subclasses, immunotherapies

## Abstract

**Background:**

This study aims to establish an N6-methyladenosine (m^6^A) RNA methylation regulators-mediated methylation model and explore its role in predicting prognostic accuracy of immune contexture and characterizations of clear cell renal cell carcinoma (ccRCC).

**Methods:**

The m^6^A modification subclasses (m^6^AMS) were identified by unsupervised cluster analysis and three clusters were determined by consensus clustering algorithm in a discovering cohort. Testing and real-world validation cohorts were used to identify predictive responses for immune checkpoint therapies (ICTs) of m^6^AMS.

**Results:**

Prognostic implications landscape of m^6^A regulators in cancers and its differential expression levels in ccRCC patients were identified. Based on discovering cohort, ccRCC were automatically divided into three m^6^AMS, and cluster 3 showed significant worse survival than cluster 1/2. Importantly, it was found that the immune checkpoint molecules expression was significantly elevated in cluster 3. Besides, m^6^A score^Low^ group (cluster 1&2) have significantly elevated TIDE score compared with m^6^A score^High^ group (cluster 3). There was conspicuous tertiary lymphoid tissue, aggressive phenotype, elevated glycolysis, expression of PD-L1, abundance of CD8^+^ T cells, CD4^+^ FOXP3^+^ Treg cells and TCRn immune cells infiltration in the high m^6^A score group. Interestingly, there are significantly increased patients with clinical benefit in m^6^A score^High^ group in 368 patients receiving ICTs from testing IMvigor210 (n = 292) and validation FUSCC (n = 55) cohorts.

**Conclusion:**

Our discovery highlights the relationship between tumor epigenetic heterogeneity and immune contexture. Immune-rejection cluster 3 has pro-tumorigenic immune infiltration, and shows significant clinical benefits for ccRCC patients receiving ICTs, enabling patient selection for future clinical treatment.

## Introduction

Renal cell carcinoma (RCC) has been widely recognized as a heterogeneous disease encompassing different histological subtypes (1). Clear cell RCC (ccRCC), the most frequent subtype (>80% of the cases) and primarily responsible for mortalities, remains one of the most burdensome genitourinary neoplasms, as its increasing incidence worldwide and a large proportion of patients present with metastasis at initial diagnosis (2). Currently, an increasing number of clinical trials demonstrated that targeted therapies, such as tyrosine-kinase inhibitors (TKIs), and immune checkpoint therapies (ICTs) have tremendously improved clinical benefits for patients with metastatic RCC (3). However, these first-line drugs are often not able to bring effective clinical relief to advanced ccRCC patients, largely due to differences in individual patient heterogeneity and lack of effective signature to predict efficacy (4–6). Therefore, it is urgently needed to reveal the intratumoral non-genomic heterogeneity, tumor microenvironment characteristics, and develop prediction model for early diagnosis, prevention and individual therapy of ccRCC.

Till now, more than 100 kinds of post-transcriptional chemical methylation have been found in organisms, and provided therapeutic potential of targeting regulators for cancer therapy (7, 8). RNA modification affects most aspects of mRNA function and reshaped the secondary structure of the RNA molecules, such as N^6^-methyladenosine (m^6^A), 2-O-dimethyladenosine (m^6^Am), N^1^-methyladenosine (m^1^A), ^5^-methylcytosine (m^5^C) and ^7^-methyl guanosine (m^7^G) (8). As the most prevalent mRNA modification, m6A is a process in which methyltransferase catalyzes the methylation modification of adenine mainly at the position of RRm6ACH (9). In 2011, He et al. revealed the reversible modification of m6A (10), mediated by an expanding list of N-methyladenosine (mA) readers, writer-complex components and eraser regulators (8). Currently, an increasing evidence demonstrated the dynamic transcriptomic mA decoration in RNA metabolism and epitranscriptome (11, 12). Therefore, the mechanism of m^6^A, the vital RNA epigenetic modification, and epigenetics cooperating to regulate gene expression is worthy of further exploration.

Recently, m^6^A are shown to be essential for tumor development and targeted drug development and outcome prediction (13, 14). The methyltransferases (m^6^A “writers”), demethylases (m^6^A “erasers”), and m^6^A “reader” proteins coordinate in the process of m6A modification. It is suggested that METTL3 could serve as a cytoplasmic m^6^A reader to promote the translation of target mRNA transcripts by interacting with the translation initiation mechanism, and promotes progressive progression in cancers (15, 16). YTH domain proteins, including YTHDF1, YTHDF2, YTHDF3, YTDHDC1 and YTHDC2, are the most reported m^6^A readers and promote the degradation of m^6^A modified mRNA and tumor progression (17–19). Additionally, splicing factor heterogeneous nuclear ribonucleoprotein A2B1 (hnRNPA2B1) significantly promotes malignant behaviors and tumorigenesis of various cancers (20, 21). Based on increasing cutting-edge scientific advances in the emerging field of phenomics, many m^6^A regulators have been identified as prognostic and showed favorable predictive value of ccRCC (22–24). Interestingly, RNA m^6^A modification could modulate the translation efficiency of lysosomal cathepsin in dendritic cells and affect a new mechanism of tumor antigen-specific T cell immune response (19). However, it is still urgently needed to comprehensively and rigorously explore the prognostic value of m^6^A regulators in ccRCC, and establish prognostic models to investigate its predictive efficiency for long-term survival and immunotherapy response.

## Materials and Methods

### Data Downloading and Extraction of 21 m^6^A Regulators

Gene expression profiles and clinical information of ccRCC were downloaded from the Cancer Genome Atlas (TCGA, https://portal.gdc.cancer.gov/). Data analysis begins with normalization of biological data. By matching the sample ID, 530 ccRCC cases with full data for gene expression and clinical information were obtained. A total of 21 m^6^A regulators were obtained from a previous study (25) and these 21 m^6^A regulators included eight writers (METTL3, RBM15, METTL14, RBM15B, KIAA1429, WTAP, CBLL1, ZC3H13), two erasers (FTO, ALKBH5) and 11 readers (YTHDF1, YTHDF2, YTHDF3, YTHDC1, YTHDC2, HNRNPC, IGF2BP1, HNRNPA2B1, LRPPRC, FMR1, ELAVL1). Gene expression of 21 m^6^A regulators in ccRCC was extracted using R software. Differentially expressed genes (DEGs) analysis was applied to evaluate the disparate expression level of m^6^A regulators between ccRCC and normal tissues adjacent to cancer from the TCGA cohort. Expression levels of 21 m^6^A regulators in pan-cancer from the TCGA cohort were also explored and compared.

### Copy Number Variation (CNV) Profiles and Drug Sensitivity of 21 m^6^A Regulators in ccRCC

We obtained the 21 m^6^A regulators genetic mutation data, transcriptome data, and clinical data of ccRCC samples from the TCGA database. Mutation data were downloaded and visualized using the “maftools” package in R software. Horizontal histogram showed the top 10 genes with higher mutation frequency in ccRCC patients. Gene Set Cancer Analysis (GSCA), an integrated genomic and immunogenomic gene set cancer analysis database, was used to predict difference of immune infiltrates between 21 m^6^A regulators CNV groups and the correlation between gene expression and the sensitivity of GDSC drugs (top 30) in pan-cancer (26).

### Correlation Analysis and Construction of m^6^A Regulators Transcriptomic Subclasses

Correlation analysis was applied to assess the association between m^6^A regulators using Spearson’s test. According to the expression of 21 m^6^A regulators, different m^6^A modification subclasses (m^6^AMS) were identified by unsupervised cluster analysis, and the patients were classified for further analysis. The number of clusters and their stability are determined by a consensus clustering algorithm. We performed the above steps using the ConsensusClusterPlus software package (27) and repeated for 1,000 times to ensure the stability of the classification. Principal component analysis was used to evaluate the differential distribution of the m^6^AMS.

### Clinical Significance and Differential Gene Expression Patterns of m^6^AMS

Kaplan–Meier method was applied to estimate the overall survival between various m^6^AMS. T stage, clinical stage, grade, gender and age were also included in comparing the m^6^AMS (N and M stages were excluded due to the incompletion of the original data). Limma R package (28) was utilized to evaluate the differential gene expression patterns between m^6^AMS. Log_2_(Fold change) >1 and *p* value <0.01 was considered statistically significant.

### Identification of Potential Alterations of Tumor Immune Microenvironment Characterizations Between the m^6^AMS Groups

The m^6^AMS were merged into two main subclasses according to similar survival benefits in different groups. A CIBERSORT (29) package is a deconvolution algorithm that uses a set of barcode gene expression values (corresponding to a “signature matrix” of 547 genes) to accurately determine the composition of immune cells in tumor sample data. In this research, a CIBERSORT was utilized to explore the potential alteration in immune cells infiltration between m^6^AMS. Functional enrichment analysis was used to find potential biological changes by using ClusterProfiler package (30). The expression of immune checkpoint-related genes between m^6^AMS was compared.

### Exploration of the Association Between the m^6^AMS and ICTs Response of ccRCC

Using bivariate analysis, we obtained a formula (m^6^A score = 1.889 ∗ HNRNPA2B1 − 0.451 ∗ ALKBH5) to identify the m^6^AMS. As there were significant changes in immune checkpoint related genes between m^6^AMS, the potential association between m^6^AMS and immune checkpoint blockade response is worth further exploration. Given that there is little available public data about ICTs’ response of large-scale ccRCC patients, we explored the potential use of the classifier in cancers cohorts. IMvigor210 is a large phase 2 trial investigating the clinical activity of PD-L1 blockade with atezolizumab in metastatic urothelial cancer. In this research, gene expression profiles of 292 pre-treatment tumors from IMvigor210 cohort were obtained to identify the potential predictive function of the classifier. Furthermore, pre-treatment tumor tissues and paired normal tissues of 76 ccRCC patients previously treated with ICTs were collected from the Fudan University Shanghai Cancer Center (FUSCC). Patients’ responses to ICTs were collected retrospectively. RT-qPCR was utilized to evaluate the relative expression of HNRNPA2B1 and ALKBH5. The m^6^A score of each sample in both cohorts was calculated and patients were stratified into different groups. Bivariate analysis was utilized to determine the association between m^6^A score and ICTs response. Receiver operating characteristic (ROC) curve was constructed to analyze the diagnostic accuracy of the genomic classifier and the area under curve (AUC) was calculated.

### Tumor Microenvironment Exploration and Opal Multispectral Imaging Between m^6^AMS of ccRCC

Tumor Immune Dysfunction and Exclusion (TIDE) was also utilized to evaluate the potential predictive ability of the genomic classifier (http://tide.dfci.harvard.edu/) (31). Tertiary lymphoid structure (TLS) was assessed using hematoxylin–eosin (HE) staining and immunohistochemistry (IHC) was utilized to evaluate the expression levels of Ki-67 (ab15580; Abcam), Glut-1 (ab115730; Abcam) and PD-L1 (ab205921; Abcam) according to procedures as previously described (32). After the slices in the repair solution (Tris-EDTA buffer, pH 9.0) are heated by an electric ceramic stove for antigen retrieval, these were rinsed with PBS (pH 7.4) for three times, and added to the prepared 3% hydrogen peroxide dropwise Block the endogenous peroxidase on the sectioned tissue, incubated at room temperature for 15 min, and then rinsed with TBS three times, each for 3 min. Goat serum was used for serum blocking for 30 min to reduce non-specific staining. CD3 (Kit-0003, Maxim, China), CD4, (RMA-0620, Maxim, China), CD8 (RMA-0514, Maxim, China), CD11C (Ab52632, Abcam), CD20 (Ab9475, Abcam), CD68 (76437, CST), CK (Kit-0009, Maxim, China), FOXP3 (98377, CST), and PD-L1 (13684, CST) antibodies were added to the slide and incubated overnight in a humidified chamber at 4°C. When using the multi-color labeling kit, the concentration of the primary antibody is 5–10 times diluted on the basis of the optimal concentration for immunohistochemistry. Then, HRP-labeled goat anti-rabbit/mouse secondary antibody was added dropwise and incubated at 37°C for 30 min. After washing with TBS, TSA-Fluorescein (Amplification Dilution dilution) is added dropwise, and incubated at 37°C for 10 min. Finally, the slices are imaged and quantitatively analyzed on a multispectral imaging system (Vectra^®^ Polaris™, Shanghai).

### Statistical Analysis

In the statistical analyses, the Wilcox test was used to compare the differences between the two groups of samples. When performing model verification in the TCGA data set, according to the correlation between different groups of m^6^ATS and patient survival, the survminer of the R package was used to determine the best cutoff value. The survival curve was analyzed by Kaplan–Meier, and the log-rank test was used to determine the significance of the difference. The receiver operating characteristic (ROC) is used to evaluate prediction sensitivity and specificity of m^6^AMS in the disease progression, and the area under the curve (AUC) is used to evaluate the specificity and sensitivity of the model.

## Results

**Figure 1:**
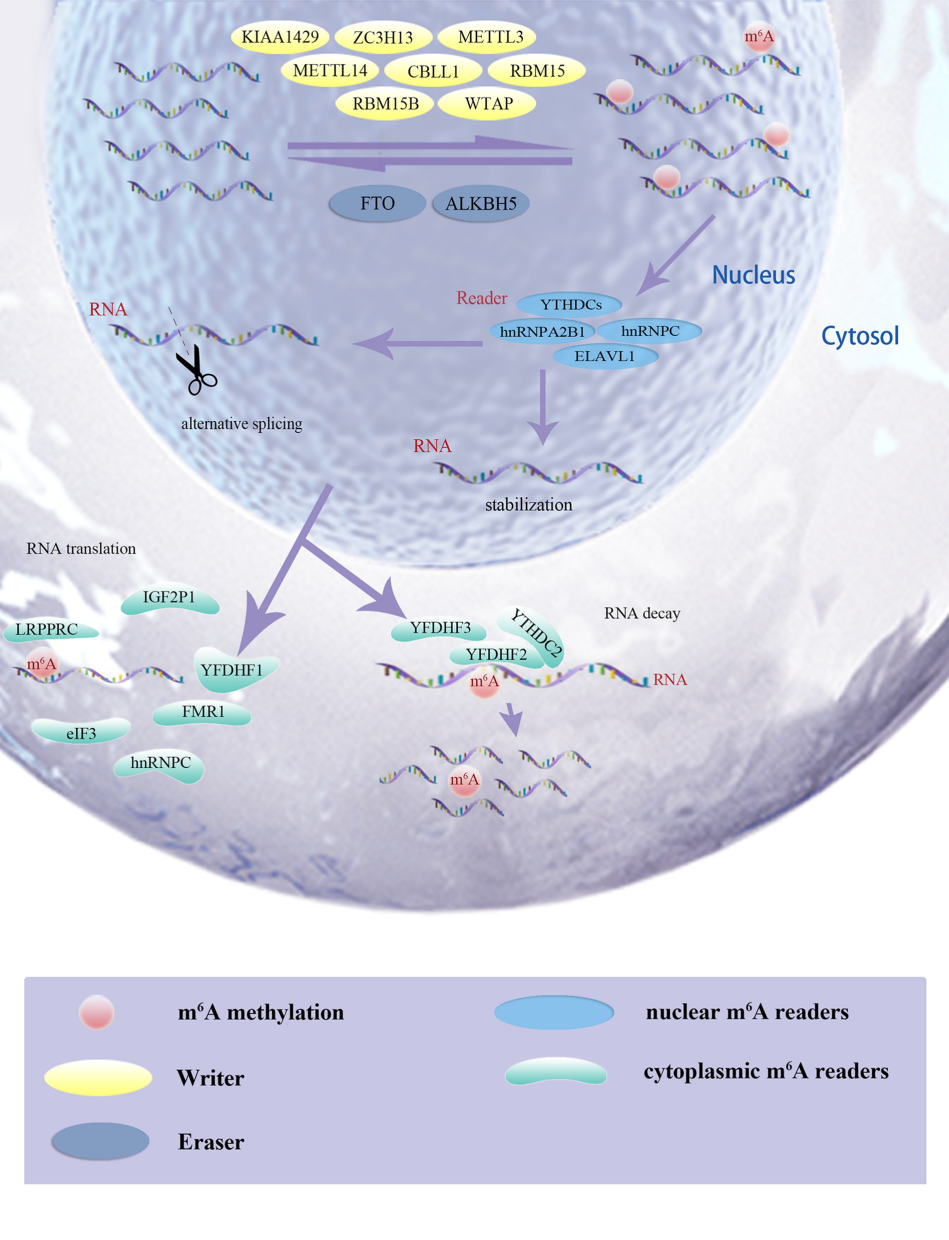
The m^6^A modification has been implicated in various cellular and physiological events, including carcinogenesis, and the mechanism of m^6^A modification was simply depicted.

The m^6^A modification has been implicated in various cellular and physiological events, including carcinogenesis, and the mechanism of m^6^A modification is simply depicted in **Figure 1**. This study was conducted in three phases. First, we assessed significant differential m^6^A regulators expression in pan-cancers and measured its novel prognostic implications in patients with ccRCC; m^6^AMS were identified using matching-learning algorithms. Second, we assessed relationships among the improved m^6^AMS and immune microenvironment characterizations of ccRCC *in silico* and *in vitro*. Third, m^6^A score was estimated in predictive responses to ICTs for ccRCC patients from two testing cohorts.

### Differential Expression Level of m^6^A Regulators Varies Between ccRCC and Normal Tissues

**Figure 2:**
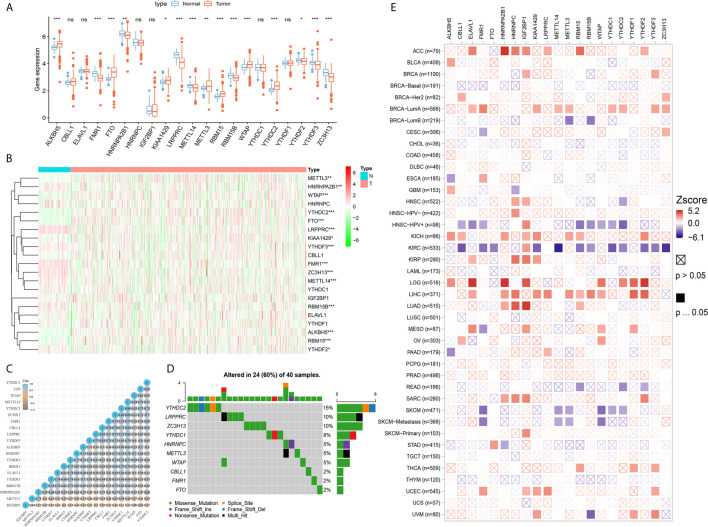
Differential expression, copy number variation landscape and prognostic implications of 21 m6A regulators in cancers. **(A)** Differential expression level of 21 m^6^A regulators in tumor and normal tissues were assessed using Student’s t test. **(B)** A heat map indicating the m^6^A modification patterns between tumor tissues and normal tissues. **(C)** A heat map of the correlation between 21 m^6^A regulators. The horizontal and vertical coordinates represent genes, and different colors represent correlation coefficients. **(D)** Oncoplot displaying the somatic landscape of 21 m6A regulator in ccRCC samples from TCGA database. Mutation information of each gene in each sample was shown in the waterfall plot, where different colors with specific annotations at the bottom meant the various mutation types. **(E)** Clinical implications of 21 m^6^A regulators expression across various cancer types was estimated in a heatmap using Cox regression methods (ns, not significant; *p  < 0.05, **p  < 0.01, ***p  < 0.001).

As shown in **Figure 2A**, expression level of 21 m^6^A regulators varies a lot between tumor and normal tissues. The expression levels of *ALKBH5*, *FTO*, *KIAA1429*, *METTL3*, *RBM15*, *WTAP*, and *YTHDC2* were significantly higher in tumor tissues, while expression levels of *FMR1*, *HNRNPA2B1*, *LRPPRC*, *METTL14*, *RBM15B*, *YTHDF2*, *YTHDF3* and *ZC3H13* were significantly decreased in tumor tissues. Next, a heatmap clearly indicated that the m^6^A modification patterns were significantly different between tumor tissues and normal tissues (**Figure 2B**). Correlation heatmap indicated that *FMR1*, *YTHDF3*, *CBLL1*, *ZC3H13*, *METTL14*, *YTHDC1*, *KIAA1429* and *LRPPRC* have strong association with others (most r^2^ >0.4; **Figure 2C**). Interestingly, Oncoplot displaying the somatic landscape of 21 m^6^A regulator in ccRCC samples from TCGA database. Mutation information of each gene in each sample was shown in the waterfall plot, where different colors with specific annotations at the bottom meant the various mutation types. It suggested that *YTHDC2*, *LRPPRC*, *ZC3H13* and *YTHDC1* have the highest mutation frequency, and nonsense mutations are the main variant classification (**Figure 2D**).

### Prognostic Implications of 21 m^6^A Regulators in Cancers

Clinical implications of 21 m^6^A regulators expression across various cancer types were estimated in a heatmap using the K–M method. The findings suggested that the elevated expression levels of m^6^A regulators, such as *CBLL1*, *METTL14*, *YTHDC1-2*, *YTHDF2-3* and *ZC3H13*, were significantly associated with favorable prognosis of ccRCC, which showed relatively converse clinical implications in patients with other cancers, such as ACC, LGG, LIHC, LUAD, SARC, etcetera (**Figure 2E**).

### Identification and Comparison of Various m^6^A Modification Patterns of ccRCC

**Figure 3:**
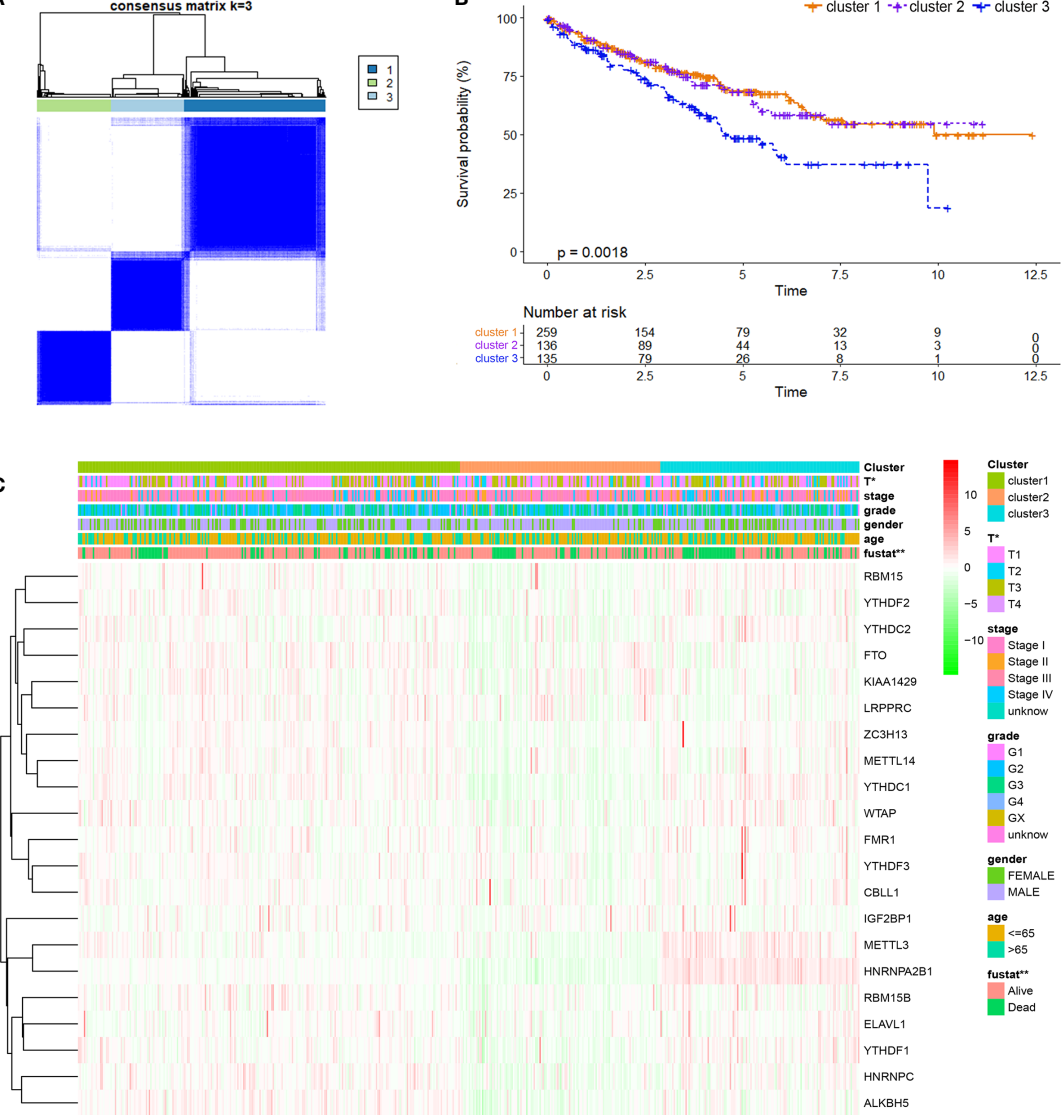
Identification and comparison of various m6A modification patterns of ccRCC. **(A)** By using ConsensusClusterPlus algorithm, ccRCC samples from TCGA cohort were automatically divided into three m^6^A modification subclasses, clusters 1/2/3. **(B)** K–M method was implemented to assess prognostic value between clusters for ccRCC patients from TCGA. **(C)** A heatmap displaying clinico-pathological implications of m^6^AMS and expression distributions of 21 m^6^A regulators (*p  < 0.05, **p  < 0.01).

By using a ConsensusClusterPlus algorithm, ccRCC samples from the TCGA cohort were automatically divided into three m^6^A modification subclasses, namely, clusters 1/2/3 (**Figure 3A**). Principal component analysis, a multivariate statistical algorithm of unsupervised clustering learning, indicated that the samples could be stratified into three clusters (**Supplementary Figure 1**). By integrating the clinical information, we found that patients in cluster 3 showed a significant worse overall survival than cluster 1/2 (*p* = 0.0018; **Figure 3B**). Besides, we investigated clinico-pathological implications of m^6^AMS and expression profiles of 21 m^6^A regulators. The heatmap indicated that the three clusters were of distinct expression pattern of 21 m^6^A regulators. Cluster 3 showed a relatively advanced AJCC stage and ISUP grade, significantly higher pathological T stage and more patient deaths. In addition, the expression of HNRNPA2B1 and METTL3 in the cluster 3 samples was higher, while the expression of ALKBH5 was lower. From another perspective, cluster 3 has the characteristics of high expression of m^6^A regulators, and clusters 1 and 2 have the characteristics of medium and lowest expressions of m^6^A regulatory factors, respectively (**Figure 3C**).

### Cluster 3 Exhibited Distinct Clinical Malignant Biological Phenotypes Than Clusters 1 & 2

**Figure 4:**
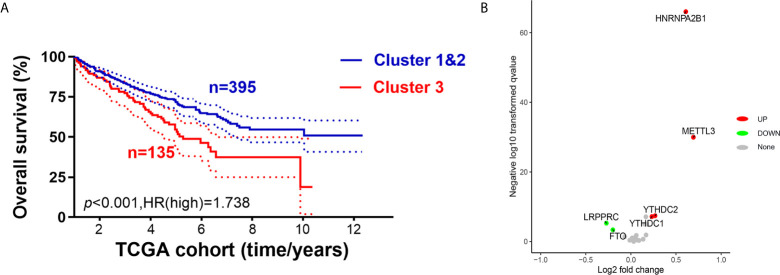
Cluster 3 exhibited distinct clinical malignant biological phenotype and suppressive immune microenvironment than cluster 1&2. **(A)** We integrated cluster 1&2 and made comparisons of survival benefits between m^6^AMS cluster 3 (n = 135) and cluster 1&2 (n = 395) using Kaplan–Meier method. **(B)** Volcano plots were constructed using fold-change values and adjusted P. The red point represents the up-regulated and the blue point indicates the down-regulated m^6^A regulators with statistical significance between cluster 3 and cluster 1&2.

As m^6^AMS cluster 1 has a similar clinical phenotype with cluster 2, we integrated clusters 1 and 2 and made comparisons of survival benefits between m^6^AMS cluster 3 (n = 135) and cluster 1&2 (n = 395). It indicated that cluster 3 was of significant worse overall survival (*p <*0.001, HR = 1.738; **Figure 4A**). In addition, we found that the expression levels of m^6^A regulators were significantly different between cluster 3 and cluster 1&2, such as significant higher expression of *HNRNPA2B1*, *METTL3*, *YTHDC1* and *YTHDC2* in cluster 3, while significantly down-regulated *LRPPRC* and *FTO* in cluster 3 (**Figure 4B**).

### Differential ccRCC Immune Microenvironment of m6AMS s and CNV Groups

**Figure 5:**
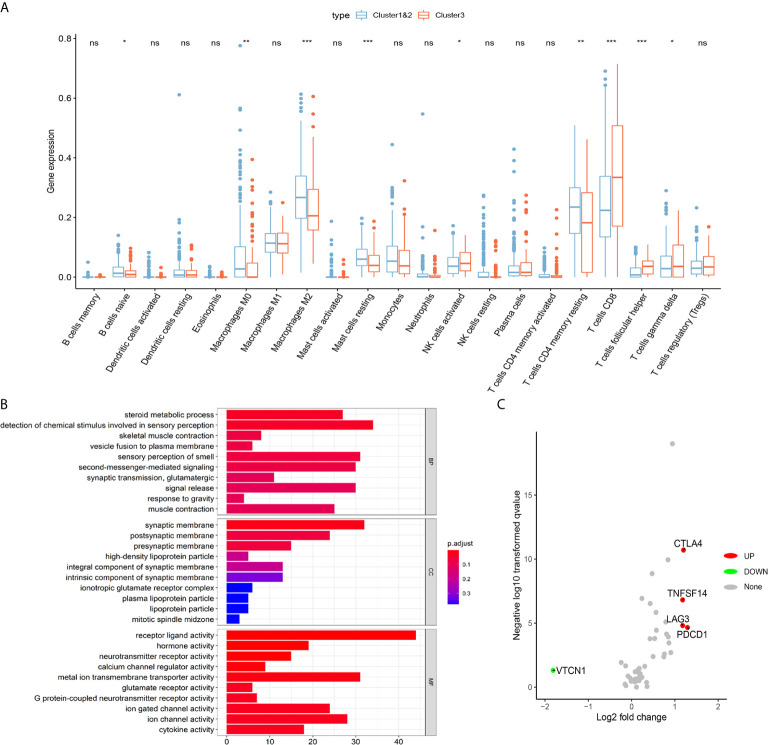
Distinct immune microenvironment of ccRCC between m6A score^Low^ and m6A score^High^ groups *in silico*. **(A)** To further explore role of m^6^AMS involved in tumor immune microenvironment characterizations, a CIBERSORT algorithm was applied to evaluate the immune cells infiltration. **(B)** Functional enrichment analyses were performed to indicate to annotate biological process, molecular function and cellular components of cluster 1&2 and cluster 3. **(C)** Volcano plots were constructed using fold-change values and adjusted P. The red point represents the up-regulated and the blue point indicates the down-regulated immune checkpoint molecules with statistical significance between cluster 3 and cluster 1&2 (ns, not significant; *p < 0.05, **p < 0.01, ***p < 0.001).

To further explore the role of m^6^AMS involved in tumor immune microenvironment characterizations, a CIBERSORT algorithm was applied to evaluate the immune cells infiltration. The abundance of naïve B cells, macrophages M0, macrophages M2, mast cells resting, T cells CD4 memory resting was relatively higher in cluster 1&2. While the abundance of NK cells activated, T cells CD8 was relatively higher in cluster 3 (**Figure 5A**). Functional enrichment analysis indicated that compared with cluster 1&2, molecular pattern of cluster 3 was mostly enriched in steroid metabolic process, synaptic membrane, receptor ligand activity, etcetera (**Figure 5B**). Importantly, it was found that the expression levels of immune checkpoint related genes, such as PDCD1, LAG3, TNFSF14 and CTLA4, were significantly elevated in cluster 3, which indicated that ccRCC samples of cluster 3 are beneficial to reshape the immune-rejection microenvironment (**Figure 5C**).

Besides, we summarized the difference of immune cells infiltrates between 21 m^6^A regulators CNV groups in **Supplementary Figure 2A**. Th1 cells was significantly amplificated in the wide-type group (n = 30) compared with the mutation group (n = 119) (*p <*0.001), while Th17, exhausted T cell and macrophages were significantly decreased in the wide-type group compared with the mutation group (*p <*0.01; **Supplementary Figure 2B**). To promote clinical translation, we explored the correlation between 21 m^6^A regulators expression and the sensitivity of GDSC drugs (top 30) in pan-cancer in **Supplementary Figure 2C**. It showed strong predictive value of anti-m^6^A regulators drugs, such as trametinib, PD-0325901, RDEA119, selumetinib and BHG712.

### Distinct Immune Microenvironment of ccRCC Between m6A score^Low^ and m6A Score^High^ Groups *In Silico* and *In Vitro*

**Figure 6:**
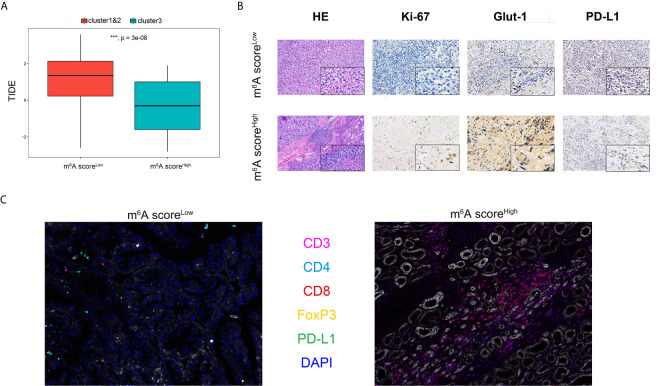
Distinct immune microenvironment of ccRCC between m6A score^Low^ and m6A score^High^ groups in *silico* and *in vitro*. **(A)** TIDE algorithm was used to indicate association between m6A score and predictive benefits for ccRCC patients receiving from ICTs. **(B)** H&E and immunohistochemistry staining were performed in different m^6^AMS clusters. **(C)** The infiltration of CD8^+^, CD4^+^, CD3^+^ and FOXP3^+^ immune cells and expression level of PD-L1 were evaluated using opal multispectral imaging (***p < 0.001).

Significantly differential immune cells infiltrations and immune check-point expression were identified *in silico*, while the alterations of various immune indicators in ccRCC immune microenvironment of different m^6^AMS clusters remain unclear. In **Figure 6A**, a TIDE algorithm indicated that m6A score^Low^ group (cluster 1&2) have significantly elevated TIDE score compared with m6A score^High^ group (cluster 3), indicating that ccRCC patients in cluster 3 were more inclined to benefit from ICTs. Next, after identification and classification of ccRCC samples from FUSCC cohort, we performed H&E and immunohistochemistry (IHC) staining analysis in different m^6^AMS clusters. It was found that there was conspicuous tertiary lymphoid tissue (TLS) in the high m^6^A score group (**Figure 6B**). IHC analysis indicated that the high m^6^A score group were of more aggressive phenotype, elevated level of glycolysis and higher expression of PD-L1, which implicated that patients in high m^6^A score group could gain more survival benefits from ICTs. Interestingly, in the m^6^A score^High^ group, the infiltration of CD8^+^ T cells, CD4^+^ FOXP3^+^ Treg cells and CD3^+^ CD4^+^ CD8^+^ TCRn immune cells were relatively higher (**Figure 6C**). Besides, the expression level of PD-L1 was also significantly elevated in the cluster 3 group, which implicated the high m^6^A score could be associated with suppressive tumor immune microenvironment and predictive response to ICTs.

### The Genomic Classifier Showed Strong Ability in Predicting ICTs Response Based on Two Testing Cohorts

**Figure 7:**
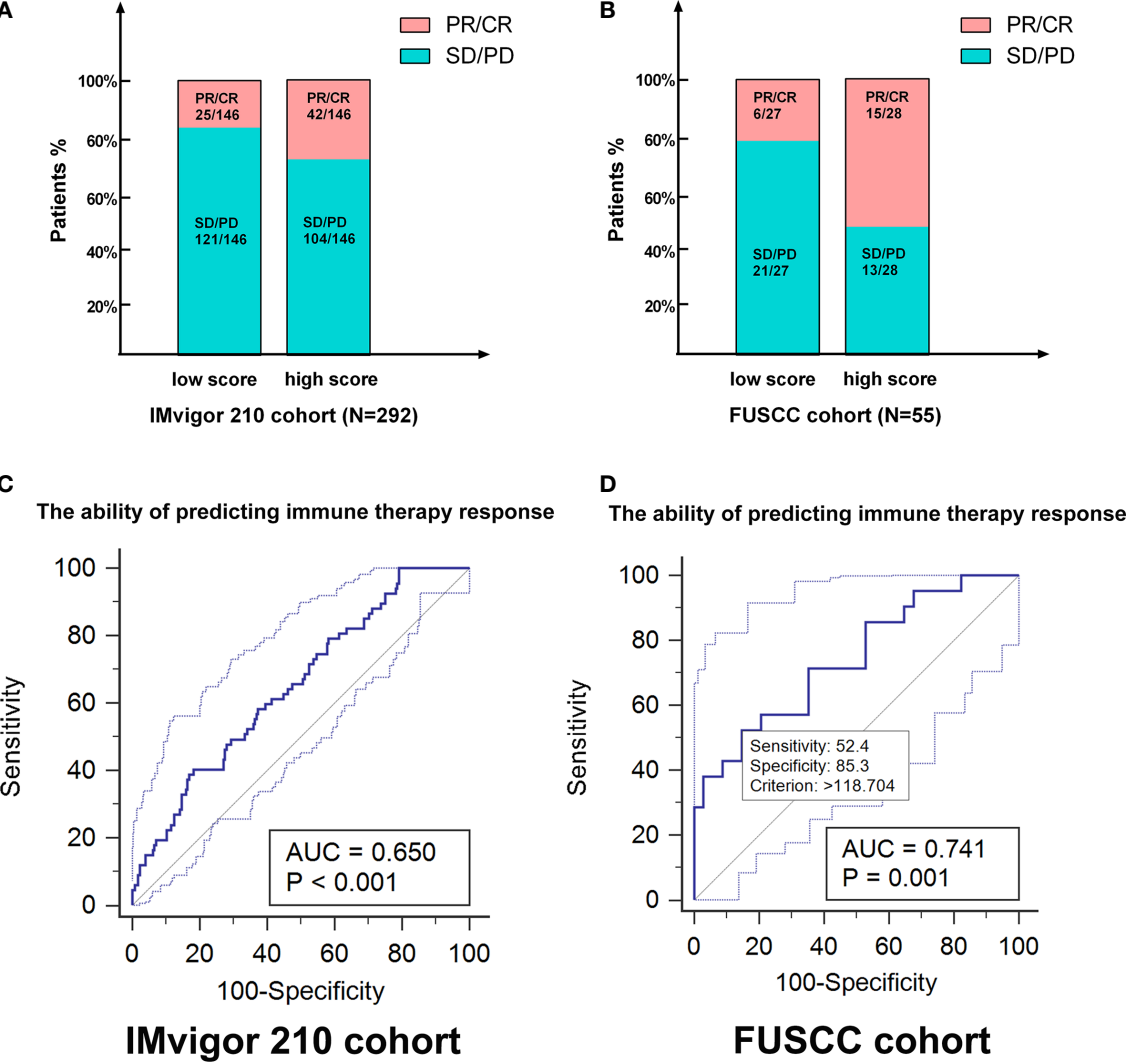
The genomic classifier showed strong ability in predicting ICTs response based on testing and validation cohorts. **(A, B)** As the cluster 3 was of significant suppressive microenvironment, we tended to construct a genomic classifier and explored its ability of predicting ICTs response. The classifier could stratify the patients into m6A scoreLow (cluster 1&2) and m6A scoreHigh (cluster 3) group. **(C, D)** ROC curves indicated that the genomic classifier has a good accuracy and stability predicting responses to ICTs (IMvigor210 cohort: AUC=0.65, p < 0.001; FUSCC cohort: AUC=0.742, p < 0.001).

As the cluster 3 was of significant suppressive microenvironment, we tended to construct a genomic classifier and explored its ability of predicting ICTs response as described in the methods. The classifier could stratify the patients into m^6^A score^Low^ (cluster 1&2) and m^6^A score^High^ (cluster 3) group. In a total of 347 patients receiving ICTs from both IMvigor210 (n = 292) and FUSCC (n = 55) cohorts, there are significantly elevated patients with clinical benefit (PR/CR) in high m^6^A score group (**Figures 7A, B**). ROC curves indicated that the genomic classifier has a good accuracy and stability predicting responses to ICTs (IMvigor210 cohort: AUC = 0.65, *p <*0.001; FUSCC cohort: AUC = 0.741, *p <*0.001; **Figures 7C, D**).

## Discussion

In the past years, many new strategies for the treatment of ccRCC have emerged, including partial or radical nephrectomy (33), targeted therapies (34), or ICTs. It is worth noting that the inherent phenotypic heterogeneity of ccRCC could aggravate tumor invasion, metastasis and drug resistance, and the non-genetic heterogeneity can be transmitted through epigenetic regulation and other mechanisms (35). Therefore, is an urgent need to construct an epigenetic genomic-based stratification model to analyze the prognosis and TME of ccRCC, thus guiding individual clinical diagnosis and treatment strategies. Based on large-scale pan cancer and ccRCC patients from public and real-world cohorts, this study first described an m^6^A regulators-mediated methylation modification model which improves the prognostic accuracy of immune contexture and characterizations in ccRCC microenvironment.

Increasing studies have shown that tumor metastasis and invasion are inseparable from the microenvironment and purity of tumor cells (36). A comprehensive analysis of the role of TME in ccRCC helps to identify the tumor immunophenotype of ccRCC, explores independent prognostic indicators and novel therapeutic targets, thereby improving the prognosis of patients and the ability to predict the effect of immunotherapy (37–39). Studies have shown that tumor purity of TME is significantly related to the clinical characteristics, genome expression, and biological characteristics of tumor patients. Previous studies also indicated that TME-based tumor purity plays a key role in tumor carcinogenesis and revealed significantly epigenetic regulators of ccRCC, opening up novel approaches in precise and personalized medicine (40).

Recent progress in the mapping of the m^6^A landscape in mRNAs, coupled with the ability to manipulate m^6^A deposition and recognition of enzymes, has begun to reveal the different molecular consequences associated with RNA methylation and its core role in different biological processes, including regulation anticancer immunity (41, 42). Despite the tremendous progress made in recent years, an understanding of how m^6^A modification affects immune phenotype is still in its infancy (19, 43, 44). In this study, differential m^6^AMS was significantly enriched in abundance of immune cells infiltration, metabolic and cancer-related immune pathways. It showed a significant immune carcinogenic status, antigen processing pathways, Macrophages M0, CD8^+^ T cells, CD4^+^ FOXP3^+^ Treg cells and TCRn cells. After analyzing the characteristics of different m^6^Acluster in different TME cells, we found that immunophenotypic classification has a strong association with suppressive tumor immune microenvironment and predictive response to ICTs for ccRCC patients. Interestingly, the m^6^AMS classifier has predictive responses to ICTs in testing cohorts, showing strong demonstrating higher predictive performance. However, more studies are needed to decipher the diversity of abnormal methylation or recognition abnormality of methylation position and tumor immunophenotype. Determining the role of various m^6^A modification modes in ccRCC microenvironment could improve our understanding of anti-tumor immune response and lay the foundation for personalized treatment of ccRCC patients (45–47).

However, this study has some limitations. First, our research failed to deeply clarify the underlying mechanism of m^6^A regulators and m^6^AMS involved in ccRCC. So, we investigated the TIME characterizations of the differential m^6^AMS on intra-tumoral heterogeneity of ccRCC *in silico* and *in vivo*. Second, although the classifier was constructed and validated using multiply datasets, due to the nature limitation of retrospective analysis, the results of our study needed to be validated in multicenter or prospective studies. Subsequently, we validated the predictive value of m^6^AMS in patients receiving ICTs or ICTs combined with from an Asian real-world cohort.

## Conclusion

This study described a m^6^A regulators-mediated methylation modification model which improves the prognostic accuracy of immune contexture and characterizations in ccRCC microenvironment. Immune-rejection cluster 3 has pro-tumorigenic immune infiltration, and shows significant clinical benefits for ccRCC patients receiving ICTs. Our discovery of the novel independent prognostic indicators in ccRCC highlights the relationship between tumor epigenetic heterogeneity and immune contexture.

## Data Availability Statement

The raw data supporting the conclusions of this article will be made available by the authors, without undue reservation.

## Ethics Statement

All of the study designs and test procedures were performed in accordance with the Helsinki Declaration II. The Ethics approval and participation consent of this study was approved and agreed by the ethics committee of Fudan University Shanghai Cancer Center (FUSCC, Shanghai, China). The patients/participants provided their written informed consent to participate in this study. Written informed consent was obtained from the individual(s) for the publication of any potentially identifiable images or data included in this article.

## Author Contributions 

Conceptualization: WX, XT, and AA. Data curation: WX, XT, WL, AA, and JS. Formal analysis: WX, WL, GW, and XT. Funding acquisition: YQ, HZ, and DY. Investigation: WX, XT, WL, AA, and JS. Methodology: WX, XT, AA, JS, and WZ. Resources: FW, GS, GW, YQ, and HZ. Software: XT, WL, WZ, and GS. Supervision: YQ, GW, HZ, and DY. Validation: WX, XT, WL, and AA. Visualization: WX, XT, AA, and JS. Roles/Writing – original draft: WX, WL, and XT. Writing – review & editing: GW, YQ, HZ, and DY. All authors contributed to the article and approved the submitted version.

## Funding

This work is supported by Grants from National Key Research and Development Project (No.2019YFC1316000), “Fuqing Scholar” Student Scientific Research Program of Shanghai Medical College, Fudan University (No. FQXZ202112B), the Natural Science Foundation of Shanghai (No.20ZR1413100) and Shanghai Municipal Health Bureau (No.2020CXJQ03) and high -level talent scientific research project of Affiliated Hospital of Youjiang Medical College for Nationalities (No. Y202011706).

## Conflict of Interest

The authors declare that the research was conducted in the absence of any commercial or financial relationships that could be construed as a potential conflict of interest.

## Abbreviations

AUC, area under curve; ccRCC, clear cell renal cell carcinoma; CNV, Copy number variation; FUSCC, Fudan University Shanghai Cancer Center; HE, hematoxylin–eosin; ICTs, immune check-point therapies; IHC, immunohistochemistry; mA, N-methyladenosine; m^6^A, N^6^-methyladenosine; m^6^AMS, m^6^A modification subclasses; RCC, Renal cell carcinoma; ROC, Receiver operating characteristic; TCGA, the Cancer Genome Atlas; TIDE, Tumor Immune Dysfunction and Exclusion; TLS, Tertiary lymphoid structure; TME, tumor microenvironment; TKIs, tyrosine-kinase inhibitors.
