# Supplementary figures and images for: m6A Regulator-Mediated Methylation Modification Model Predicts Prognosis, Tumor Microenvironment Characterizations and Response to Immunotherapies of Clear Cell Renal Cell Carcinoma
Source: Front Oncol. 2021 Jul 6;11:709579. doi: 10.3389/fonc.2021.709579 (PMC8290143; doi:10.3389/fonc.2021.709579)

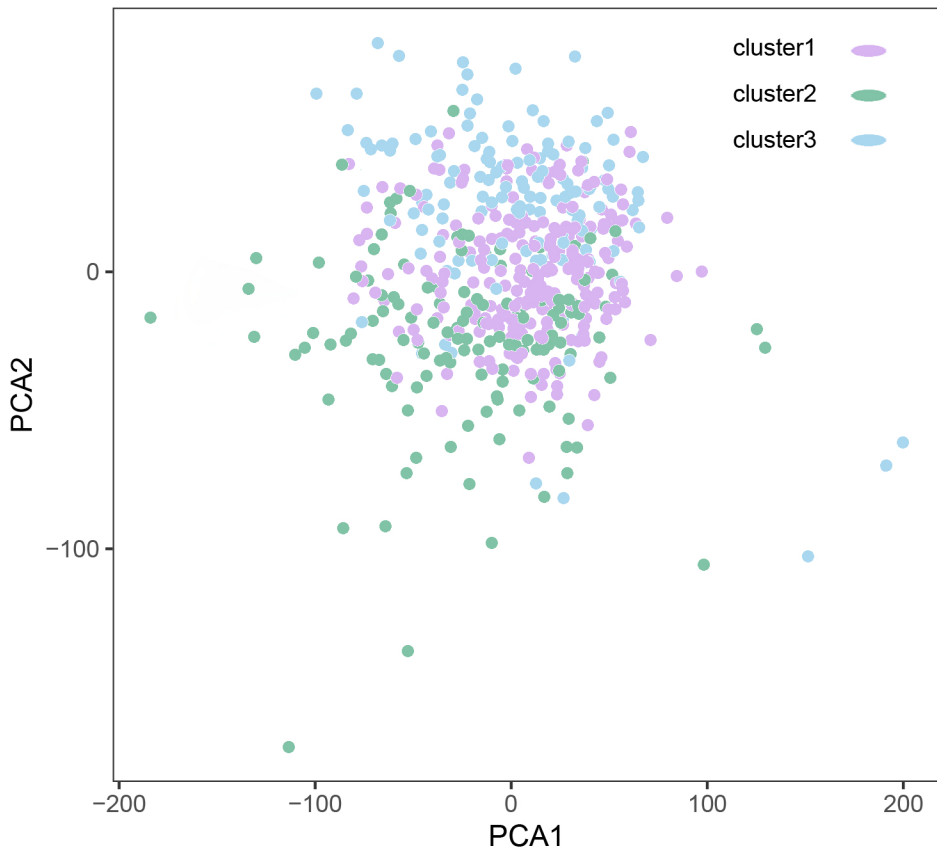

Supplement: Supplementary file 1 [file DataSheet_1.pdf]

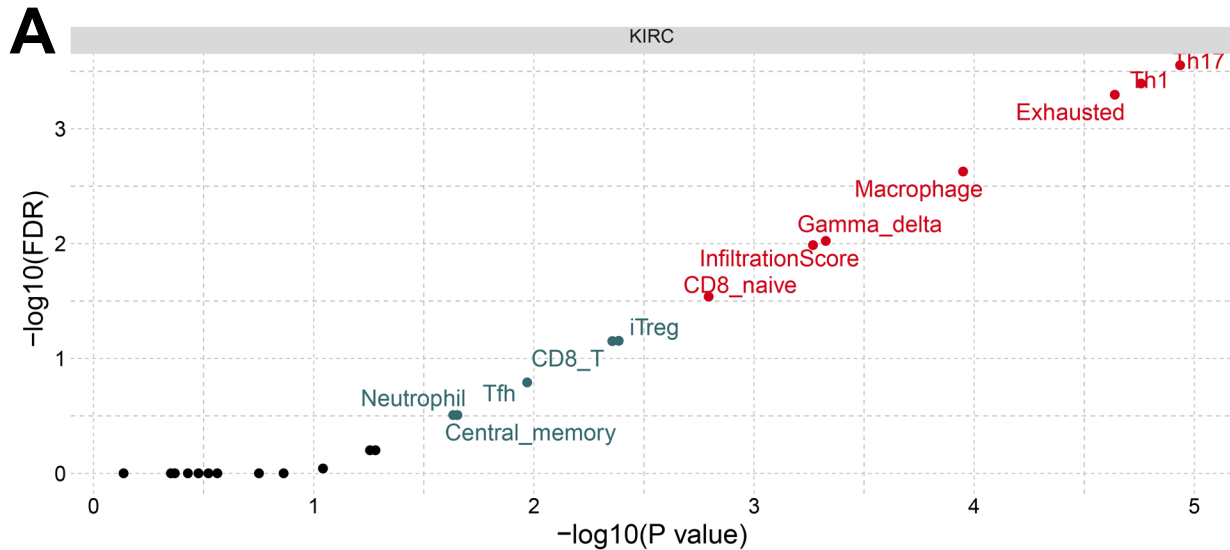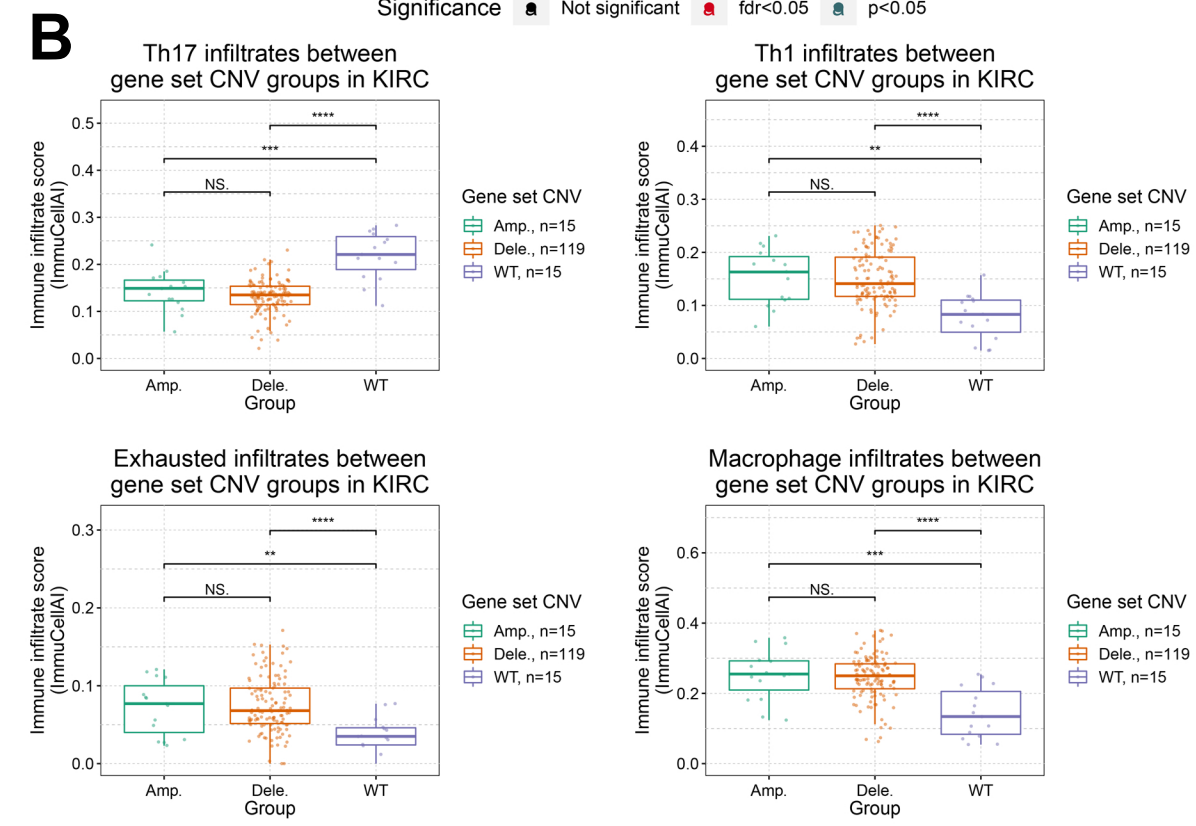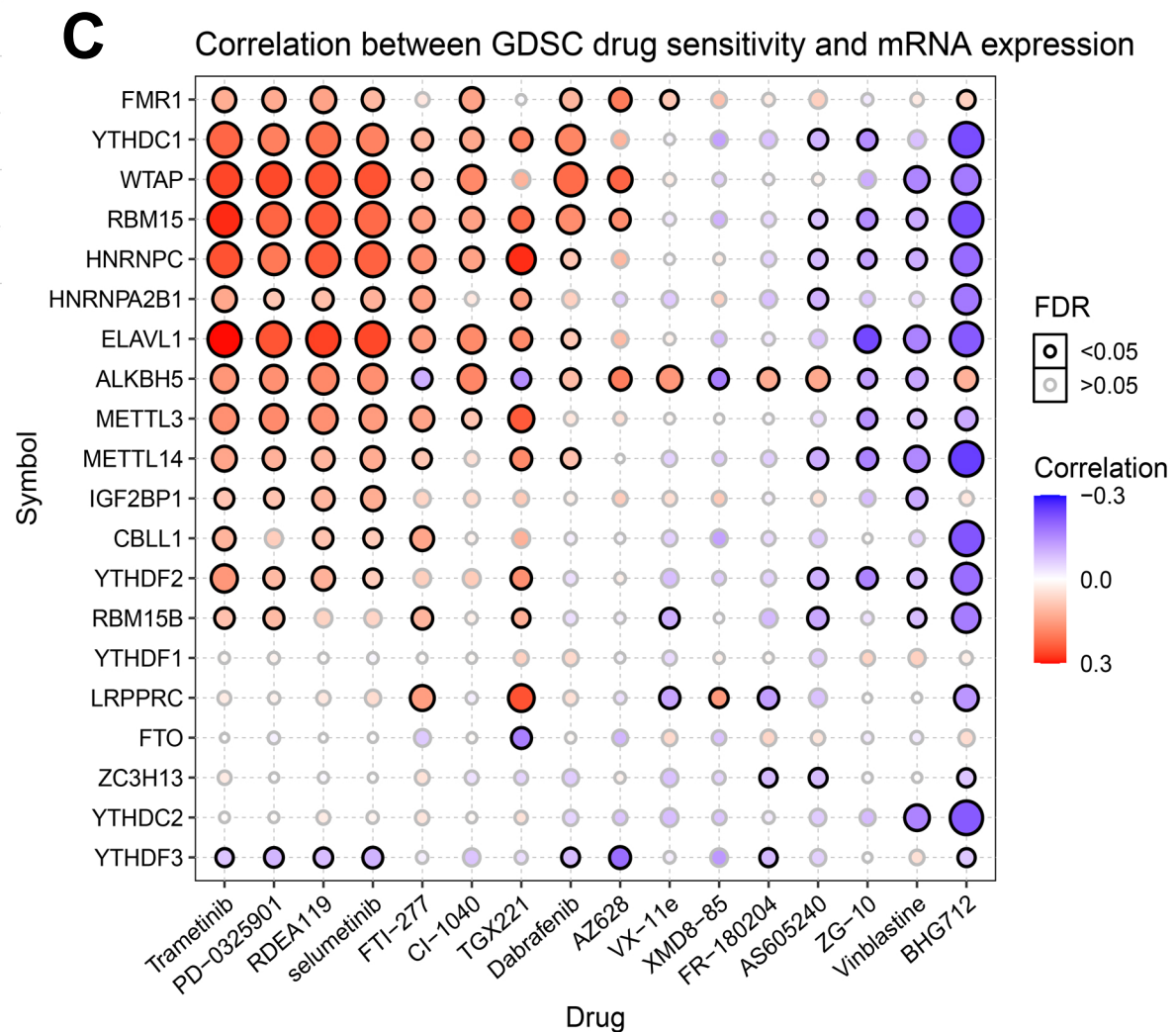

Supplement: Supplementary file 2 [file DataSheet_2.pdf]
